# A Strand-Specific Quantitative RT-PCR Method for Detecting vRNA, cRNA, and mRNA of H7N9 Avian Influenza Virus in a Mouse Model

**DOI:** 10.3390/v17071007

**Published:** 2025-07-17

**Authors:** Bo Wang, Guangwen Wang, Yi-han Wang, Xuwei Liu, Manman Li, Huihui Kong, Hualan Chen, Li Jiang, Chengjun Li

**Affiliations:** State Key Laboratory for Animal Disease Control and Prevention, Harbin Veterinary Research Institute, Chinese Academy of Agricultural Sciences, Harbin 150069, China; wangbocaas@163.com (B.W.); wangguangwen@caas.cn (G.W.); wangyihan741@126.com (Y.-h.W.); jlxuwei11@163.com (X.L.); leemanmanya@163.com (M.L.); konghuihui@caas.cn (H.K.); chenhualan@caas.cn (H.C.)

**Keywords:** H7N9, avian influenza virus, qRT-PCR, replication kinetics

## Abstract

Avian influenza virus (AIV) remains a persistent threat to both the poultry industry and human health. Among the AIV subtypes posing public health threats, H7N9 AIV is responsible for five epidemic waves of human infection in China. Here, a detection system based on a mouse model was established, which can simultaneously and quantitatively analyze the dynamic changes in the viral genomic RNA (vRNA), complementary RNA (cRNA), and messenger RNA (mRNA) of H7N9 AIV by using reverse transcription primers with tag sequences to reverse transcribe the three species of RNAs into corresponding cDNA templates, which are then absolutely quantified using the TaqMan quantitative PCR method. This system specifically targets the PB2 and NA genes and, for the first time, enables a spatiotemporal analysis of all three viral RNA species within an animal model. Our results revealed that H7N9 AIV exhibits characteristic replication kinetics, with all three species of viral RNAs showing a rapid increase followed by a certain degree of decline. This system offers a powerful tool for us to further advance our understanding of the replication dynamics of AIV in mice.

## 1. Introduction

Avian influenza viruses (AIVs) persistently cross the species barrier to infect and even kill humans, with the H7N9 AIV as a prominent representative. In 2013, H7N9 AIV jumped into humans, leading to 1568 confirmed cases and 616 deaths [1,2]. The E627K mutation in the PB2 protein was not detected in any of the H7N9 viruses isolated from poultry; however, about 80% of H7N9 viruses isolated from humans contain PB2 E627K [3]. It was revealed that the acquisition of the mammalian adaptive PB2 E627K substitution was a key determinant of viral pathogenicity [4,5,6,7,8,9]. This mutation dramatically enhances viral replication in mammalian hosts and is closely associated with severe disease outcomes [1,10,11,12]. Although such findings have advanced our understanding on the molecular basis of enhanced pathogenicity of AIVs in mammals, the replication dynamics of AIVs in mammalian hosts remain poorly characterized.

The replication of influenza virus in host cells involves the synthesis of viral genomic RNA (vRNA), complementary RNA (cRNA), and messenger RNA (mRNA) [13,14,15]. At present, the strand-specific quantitative reverse transcription PCR (qRT-PCR) is in use to analyze viral replication kinetics by detecting vRNA, cRNA, and mRNA, but this technique is limited to cultured cells [16]. An alternative method for detecting vRNA, cRNA, and mRNA is the influenza virus enumerator of RNA transcripts (INVERT) system [17]. While it is capable of identifying all three viral RNA species, it has not yet been applied to animals. In this study, we established a method for the characterization of viral replication kinetics based on a mouse model. By optimizing the strand-specific qRT-PCR technique, which involves changing the tag sequence of the RT primers and qPCR primer-probe sets, we achieved a simultaneous quantification of the three viral RNA species during H7N9 AIV infection in mice. This approach provides a novel tool to investigate the replication dynamics of the influenza virus in mammalian hosts.

## 2. Materials and Methods

### 2.1. Virus and Facilities

The H7N9 AIV strain, A/pigeon/Shanghai/S1421/2013 (PG/S1421), exhibiting a low pathogenicity in mice [1], was propagated in embryonated specific-pathogen-free (SPF) chicken eggs as previously described [18]. All experiments involving infectious H7N9 AIV were performed in the enhanced animal biosafety level 3 laboratory in the Harbin Veterinary Research Institute (HVRI) of the Chinese Academy of Agricultural Sciences (CAAS).

### 2.2. Mouse Study

Five groups of 6-week-old female C57BL/6 mice (three mice/group) were lightly anesthetized with CO_2_ and intranasally infected with 10^6.0^ 50% egg infectious dose (EID_50_) of H7N9 AIV [19]. Three mice were euthanized at 6, 12, 24, 36, and 48 h post infection (p.i.), and their lungs were collected, cut into small pieces, and preserved in RNAprotect Tissue Reagent (Cat. No. 76106; QIAGEN, Hilden, Germany) until the total RNA was extracted. The protocols were approved by the Committee on the Ethics of Animal Experiments of the HVRI of the CAAS (No. 231017-03-GJ).

### 2.3. RNA Extraction

The lungs of mice, preserved at −20 °C in RNAprotect Tissue Reagent, were washed once with RNase-free water and homogenized in Buffer RLT (a component of the RNeasy Mini Kit, Cat. No. 74106; QIAGEN, Hilden, Germany) using the FastPrep-24 5G system (MP Biomedicals, Irvine, CA, USA). The total RNA of the homogenate was extracted using the RNeasy Mini Kit.

### 2.4. In Vitro Generation of RNA

The DNA sequences corresponding to vRNA, cRNA, and mRNA of the PB2 and NA genes of H7N9 AIV were cloned into the pMD18-T vector (Cat. No. 6011; Takara Bio, Kusatsu, Shiga, Japan). Full-length double-stranded DNA products corresponding to each viral RNA species and containing the T7 promoter were amplified using recombinant vectors as templates with EasyTaq DNA Polymerase (Cat. No. AP111-03; TransGen Biotech, Beijing, China) and subsequently purified with the GeneJET Gel Extraction Kit (Cat. No. K0692; Thermo Fisher Scientific, Waltham, MA, USA). The vRNA, cRNA, and mRNA of the PB2 and NA genes of H7N9 AIV were transcribed in vitro from double-stranded DNA products using the RiboMax Large Scale RNA Production System (Cat. No. P1320; Promega, Madison, WI, USA) [20]. The RNAs transcribed in vitro were concentrated and purified using the RNeasy Mini Kit and quantified using the NanoDrop spectrophotometer (Thermo Fisher Scientific, Waltham, MA, USA).

### 2.5. Hot-Start Reverse Transcription with a Tagged Primer

To obtain the cDNA of the vRNA, cRNA, and mRNA of the PB2 and NA genes of H7N9 AIV, the RNA samples (2 μg in a volume of 5 μL) were first heated at 65 °C for 5 min, and the tagged primers (the tag sequences were derived from the soybean genome; Supplementary Information Tables S1 and S2) of the three types of viral RNAs were then individually added to configure the system (Supplementary Information Tables S3 and S4) and reversely transcribe the RNAs using the HiScript II 1st Strand cDNA Synthesis Kit (Cat. No. R212-02; Vazyme, Nanjing, China). Strand-specific RT was performed at 55 °C for 45 min for vRNA, and at 60 °C for 45 min for cRNA and mRNA, followed by incubation at 85 °C for 2 min and a hold at 4 °C.

### 2.6. TaqMan qRT-PCR

The TaqMan qRT-PCR was performed with the Premix Ex Taq (Probe qPCR) (Cat No. RR390A; Takara, Kusatsu, Shiga, Japan) on a QuantStudio 5 Real-Time PCR System (Cat. No. A58986; Applied Biosystems, Waltham, MA, USA). The details of the primer–probe sets used for the amplification of the cDNA generated from PB2 or NA vRNA, cRNA, and mRNA are provided in Supplementary Information Tables S1 and S2. All probes were labeled with FAM at the 5′ end and MGB quencher at the 3′ end. The tenfold serial dilutions of cDNA derived from the transcribed viral RNAs (10^2^–10^9^ copies/2 μL for PB2 vRNA and mRNA, 10^3^–10^9^ copies/2 μL for PB2 cRNA, 10^2^–10^7^ copies/2 μL for NA vRNA and cRNA, and 10^2^–10^9^ copies/2 μL for NA mRNA) were used to generate standard curves.

### 2.7. Data Acquisition and Analysis

The Ct values and corresponding RNA quantities from the qRT-PCR assays were obtained using the QuantStudio Design & Analysis Software (version 1.4.2). The software generated standard curves based on the serial dilutions of reference templates and calculated the absolute quantities of viral RNAs in each sample accordingly. The quantitative data were presented as the means ± SD of the three biological replicates.

## 3. Results

### 3.1. Detection of PB2 and NA vRNA, cRNA, and mRNA of H7N9 AIV in Mice

**Figure 1:**
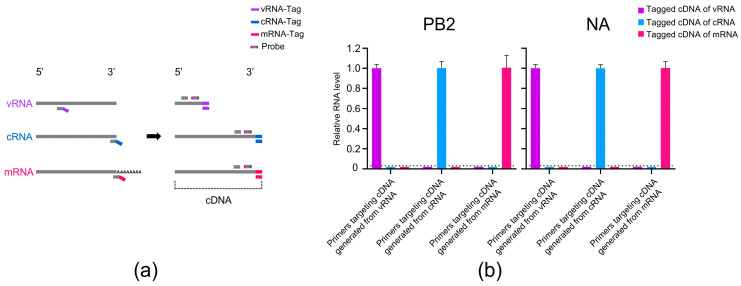
Cross-amplification of cDNAs derived from vRNA, cRNA, and mRNA of H7N9 AIV using three primer pairs by TaqMan qRT-PCR. (**a**) Schematic diagram of the TaqMan qRT-PCR method for detecting vRNA, cRNA, and mRNA of H7N9 AIV. Strand-specific RT was performed using primers containing the vRNA-tag (purple), cRNA-tag (light blue), or mRNA-tag (rose red) to generate three distinct types of cDNA. Each tag sequence, together with a gene-specific primer, formed a primer pair for amplification. Fluorescent probes were added, and quantification was conducted using TaqMan qRT-PCR. (**b**) Strand-specific cDNAs of H7N9 AIV were generated from the total RNA extracted from the mouse lungs using primers containing tag sequences, each of which contained a 20-nucleotide sequence unrelated to the influenza virus at the 5′ end. The tagged cDNA was amplified by TaqMan qRT-PCR using the tag portion of the cDNA as the forward primer, a segment-specific oligonucleotide as the reverse primer, and a corresponding probe. Each primer pair was considered as an analytical group, and the Ct values were normalized to the average Ct value of the corresponding targeted cDNA to calculate the relative RNA levels within the group. Values below the dashed line indicate undetectable fluorescence or the absence of amplification curves.

To enable detection of the vRNA, cRNA, and mRNA of H7N9 AIV in mice, we optimized the strand-specific qRT-PCR method by Kawakami et al. [16] by replacing the primers and using TaqMan qRT-PCR for amplification. This modification allowed the detection of specific fluorescence signals using the total RNA extracted from the mouse lung, as illustrated in Figure 1a. The total RNA from the lungs of H7N9 AIV-infected mice at 36 h p.i. was subjected to a hot-start RT to generate the tagged cDNA corresponding to the three RNA species of the PB2 and NA genes. The tagged cDNA was then amplified by TaqMan qRT-PCR to detect the fluorescence signals. We found that the vRNA TaqMan qRT-PCR system produced fluorescence signals only when the cDNA generated with the primer containing the vRNA-tag was used as template, but not when the cDNA generated with the primers containing the cRNA-tag or mRNA-tag was used. A similar specificity was observed for the detection of cRNA and mRNA (Figure 1b). These findings demonstrate that the optimized TaqMan qRT-PCR method can specifically detect the vRNA, cRNA, and mRNA of the PB2 and NA genes of H7N9 AIV in mice.

### 3.2. Absolute Quantification of Viral RNAs of H7N9 AIV in Mouse Lung Tissue

**Figure 2:**
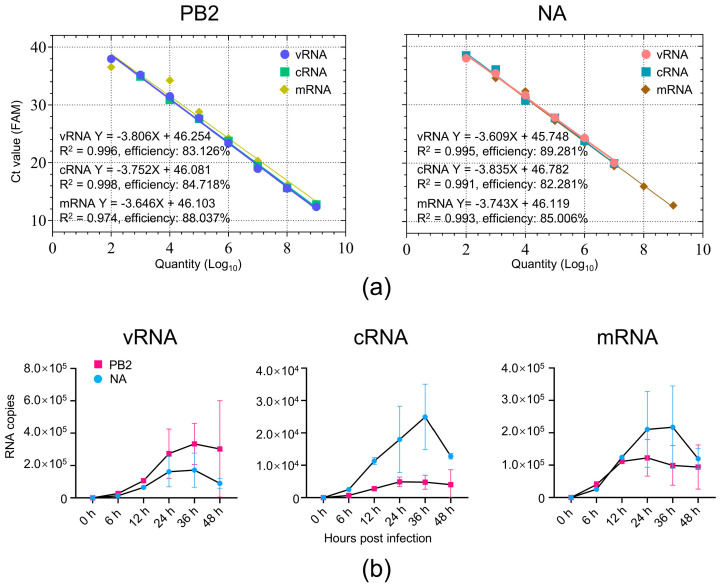
TaqMan qRT-PCR assay for the quantification of the viral RNAs of PB2 and NA genes of H7N9 AIV in mice. (**a**) Standard curves for the three types of RNAs of PB2 and NA genes, generated by plotting the Ct values against the copy numbers of the input transcribed RNA. Tenfold serial dilutions of the cDNA derived from the transcribed viral RNAs were used to generate standard curves. The standard curve parameters and amplification efficiency are shown in the lower left corner. (**b**) Synthesis kinetics of vRNA, cRNA, and mRNA of the PB2 and NA genes in the lungs of H7N9 AIV-infected mice. RNA copy numbers were quantified by TaqMan qRT-PCR using the standard curves of the cDNA derived from the transcribed viral RNA. Values represent viral RNA copies in 2 μg of the total RNA of the mouse lungs. Error bars indicate the standard deviation of three biological replicates.

To evaluate the sensitivity and amplification efficiency of the established primer–probe sets, we first transcribed full-length vRNA, cRNA, and mRNA transcripts of the viral PB2 and NA genes in vitro. The copy numbers of the transcribed RNAs were calculated based on their molecular weights. A series of tenfold serial dilutions were prepared to generate standard curves using the corresponding Ct values. The assay exhibited high reproducibility among replicates, and the detection limits for each strand-specific assay were as follows: 46.254 copies for PB2 vRNA, 46.081 copies for PB2 cRNA, 46.103 copies for PB2 mRNA, 45.748 copies for NA vRNA, 46.782 copies for NA cRNA, and 46.119 copies for NA mRNA. All reactions yielded amplification efficiencies greater than 82%, with strong linear correlations across the standard curves (Figure 2a and Figure S1). These results demonstrate that the optimized TaqMan qRT-PCR method is suitable for the quantitative detection of target viral RNAs in mouse lung tissue.

To validate the applicability of the established method, we quantified viral RNA levels in the lungs of the H7N9 AIV-infected mice. For each sample, 2 μg of the total RNA was used for the strand-specific RT to generate tagged cDNA templates. A quantitative detection of vRNA, cRNA, and mRNA for the PB2 and NA genes was performed using the TaqMan qRT-PCR, with the standard curves generated simultaneously, and the quantification results were expressed as RNA copies. As shown in Figure 2b, at 6 h p.i., the mean copies of vRNA, cRNA, or mRNA were numerically similar between PB2 and NA. However, a numerical separation in the mean copies of the viral RNAs between the PB2 and NA genes gradually emerged, with the cRNA from 12 h p.i. and the vRNA and mRNA from 24 h p.i., indicating a divergent transcriptional or replicative efficiency among different viral gene segments during the progression of the virus infection. Before 24 h p.i., the intra-group variability in the viral RNA levels was minimal, but became increasingly apparent starting from 24 h p.i., suggesting that viral replication became more variable among the hosts. In addition, the overall trend of the levels of the three viral RNA species exhibited a rapid increase up to 24 or 36 h p.i., followed by a certain degree of decline afterwards.

Together, these findings demonstrate that the optimized TaqMan qRT-PCR assay enables the quantitative analysis of the viral RNA species of H7N9 AIV in mice and effectively captures the replication dynamics of the virus in mammalian hosts.

## 4. Discussion

Strand-specific quantitative RT-PCR and the INVERT system have been used to distinguish and quantify influenza vRNA, cRNA, and mRNA, offering valuable insights into viral replication [16,17]. However, both methods have only been applied in cultured cells, and the methods of analyzing these RNA species in animals remain lacking. Here, we developed a qRT-PCR method to detect three types of viral RNAs in mouse lungs, providing a novel tool to investigate the replication dynamics of the influenza virus in animals.

H7N9 AIV exhibits a pronounced propensity to acquire the adaptive PB2 E627K mutation in mammalian hosts, which greatly enhances its replication efficiency [21,22]. To precisely assess the replication dynamics of H7N9 AIV in mice, the present study selected PB2 as an internal gene and NA as a surface glycoprotein gene for a quantitative viral RNA analysis.

The influenza virus shows less variation in replication among different biological replicates when cultured and propagated in vitro [23]. In contrast, viral replication in vivo displays marked heterogeneity [24,25]. In this study, we observed that the viral RNA levels in the mouse lungs exhibited relatively minimal variation before 24 h p.i. but showed pronounced intra-group variations thereafter. These findings indicate that the 24 h time point represents a critical window of the virus–host interaction, at which antiviral intervention may offer significant clinical benefits.

We found that from 6 to 48 h p.i., changes in cRNA and mRNA levels generally paralleled those of the vRNA. Notably, the average level of PB2 cRNA was consistently lower than that of NA cRNA, whereas the average level of PB2 vRNA was consistently higher than that of NA vRNA. This pattern suggests that different viral genes may differ in their priority of synthesizing specific RNA species during virus infection in vivo.

We observed that the three types of viral RNAs exhibited a rapid increase up to 24 or 36 h p.i., followed by a moderate decline. This pattern is consistent with a typical viral replication cycle. The subsequent reduction in viral RNA levels may stem from the activation of innate immune responses that suppress viral gene synthesis, intracellular antiviral mechanisms that degrade viral proteins and hinder further replication, or viral adaptation processes that promote a dynamic equilibrium between the host and the virus.

Although the present study has advantages in detecting the vRNA, cRNA, and mRNA of H7N9 AIV in mice, some limitations still need to be considered. The study focuses on two representative genes, PB2 and NA, which may not reflect the synthesis profile of other viral segments, such as NS, the one encoding NS1 that is linked to immune evasion. Future research should focus on a genome-wide analysis of the influenza virus’ RNA dynamics in the host to provide more comprehensive insights into viral replication. In addition, H7N9-infected patients exhibited increased cytokine and chemokine levels in their lungs [1]. This study does not examine the link between viral RNA levels and cytokine/chemokine levels. Future research should integrate the viral replication analysis with a transcriptomic or proteomic analysis of the host response to better understand virus–host interactions.

In summary, we developed a strand-specific TaqMan qRT-PCR method for precise quantification of the vRNA, cRNA, and mRNA of the influenza virus. Applied in mice, this method enabled an analysis of the replication dynamics of H7N9 AIV and provided a key tool for understanding the spatiotemporal regulation of viral replication in the host.

## Data Availability

The original contributions presented in this study are included in the article/Supplementary Material. Further inquiries can be directed at the corresponding authors.

## Supplementary Materials

The following supporting information can be downloaded at: https://www.mdpi.com/article/10.3390/v17071007/s1, Figure S1: Amplification curves of tenfold serial dilutions of cDNA derived from transcribed viral RNA; Table S1: Sequences of TaqMan qRT-PCR primer and probe sets for detection of vRNA, cRNA, and mRNA of the PB2 gene; Table S2: Sequences of TaqMan qRT-PCR primer and probe sets for detection of vRNA, cRNA, and mRNA of the NA gene; Table S3: Strand-specific reverse transcription system of PB2 or NA vRNA; Table S4: Strand-specific reverse transcription system of PB2 or NA cRNA and mRNA.

## References

[1] Wan X., Li J., Wang Y., Yu X., He X., Shi J., Deng G., Zeng X., Tian G., Li Y. (2022). H7N9 virus infection triggers lethal cytokine storm by activating gasdermin E-mediated pyroptosis of lung alveolar epithelial cells. Natl. Sci. Rev..

[2] Shi J., Zeng X., Cui P., Yan C., Chen H. (2023). Alarming situation of emerging H5 and H7 avian influenza and effective control strategies. Emerg. Microbes Infect..

[3] Shi J., Deng G., Kong H., Gu C., Ma S., Yin X., Zeng X., Cui P., Chen Y., Yang H. (2017). H7N9 virulent mutants detected in chickens in China pose an increased threat to humans. Cell Res..

[4] de Jong R.M., Stockhofe-Zurwieden N., Verheij E.S., de Boer-Luijtze E.A., Ruiter S.J., de Leeuw O.S., Cornelissen L.A. (2013). Rapid emergence of a virulent PB2 E627K variant during adaptation of highly pathogenic avian influenza H7N7 virus to mice. Virol. J..

[5] Yamayoshi S., Fukuyama S., Yamada S., Zhao D., Murakami S., Uraki R., Watanabe T., Tomita Y., Neumann G., Kawaoka Y. (2015). Amino acids substitutions in the PB2 protein of H7N9 influenza A viruses are important for virulence in mammalian hosts. Sci. Rep..

[6] Lee C.Y., An S.H., Kim I., Go D.M., Kim D.Y., Choi J.G., Lee Y.J., Kim J.H., Kwon H.J. (2017). Prerequisites for the acquisition of mammalian pathogenicity by influenza A virus with a prototypic avian PB2 gene. Sci. Rep..

[7] Qin J., Peng O., Shen X., Gong L., Xue C., Cao Y. (2019). Multiple amino acid substitutions involved in the adaption of three avian-origin H7N9 influenza viruses in mice. Virol. J..

[8] Liu W.J., Li J., Zou R., Pan J., Jin T., Li L., Liu P., Zhao Y., Yu X., Wang H. (2020). Dynamic PB2-E627K substitution of influenza H7N9 virus indicates the in vivo genetic tuning and rapid host adaptation. Proc. Natl. Acad. Sci. USA.

[9] Zhu W., Li L., Yan Z., Gan T., Li L., Chen R., Chen R., Zheng Z., Hong W., Wang J. (2015). Dual E627K and D701N mutations in the PB2 protein of A(H7N9) influenza virus increased its virulence in mammalian models. Sci. Rep..

[10] Wang Z., Zhang A., Wan Y., Liu X., Qiu C., Xi X., Ren Y., Wang J., Dong Y., Bao M. (2014). Early hypercytokinemia is associated with interferon-induced transmembrane protein-3 dysfunction and predictive of fatal H7N9 infection. Proc. Natl. Acad. Sci. USA.

[11] Zhang H., Li X., Guo J., Li L., Chang C., Li Y., Bian C., Xu K., Chen H., Sun B. (2014). The PB2 E627K mutation contributes to the high polymerase activity and enhanced replication of H7N9 influenza virus. J. Gen. Virol..

[12] Shi J., Deng G., Ma S., Zeng X., Yin X., Li M., Zhang B., Cui P., Chen Y., Yang H. (2018). Rapid Evolution of H7N9 Highly Pathogenic Viruses that Emerged in China in 2017. Cell Host Microbe.

[13] Dou D., Revol R., Ostbye H., Wang H., Daniels R. (2018). Influenza A Virus Cell Entry, Replication, Virion Assembly and Movement. Front. Immunol..

[14] Dawson A.R., Wilson G.M., Coon J.J., Mehle A. (2020). Post-Translation Regulation of Influenza Virus Replication. Annu. Rev. Virol..

[15] Carter T., Iqbal M. (2024). The Influenza A Virus Replication Cycle: A Comprehensive Review. Viruses.

[16] Kawakami E., Watanabe T., Fujii K., Goto H., Watanabe S., Noda T., Kawaoka Y. (2011). Strand-specific real-time RT-PCR for distinguishing influenza vRNA, cRNA, and mRNA. J. Virol. Methods..

[17] Phan T., Fay E.J., Lee Z., Aron S., Hu W.S., Langlois R.A. (2021). Segment-specific kinetics of mRNA, cRNA and vRNA accumulation during influenza infection. J. Virol..

[18] Zhang Q., Shi J., Deng G., Guo J., Zeng X., He X., Kong H., Gu C., Li X., Liu J. (2013). H7N9 influenza viruses are transmissible in ferrets by respiratory droplet. Science.

[19] Hou Y., Deng G., Cui P., Zeng X., Li B., Wang D., He X., Yan C., Zhang Y., Li J. (2024). Evolution of H7N9 highly pathogenic avian influenza virus in the context of vaccination. Emerg. Microbes Infect..

[20] Feng X., Wang Z., Shi J., Deng G., Kong H., Tao S., Li C., Liu L., Guan Y., Chen H. (2016). Glycine at Position 622 in PB1 Contributes to the Virulence of H5N1 Avian Influenza Virus in Mice. J. Virol..

[21] Chan L.L., Bui C.T., Mok C.K., Ng M.M., Nicholls J.M., Peiris J.S., Chan M.C., Chan R.W. (2016). Evaluation of the human adaptation of influenza A/H7N9 virus in PB2 protein using human and swine respiratory tract explant cultures. Sci. Rep..

[22] Lee C.Y., An S.H., Choi J.G., Lee Y.J., Kim J.H., Kwon H.J. (2020). Rank orders of mammalian pathogenicity-related PB2 mutations of avian influenza A viruses. Sci. Rep..

[23] Frensing T., Kupke S.Y., Bachmann M., Fritzsche S., Gallo-Ramirez L.E., Reichl U. (2016). Influenza virus intracellular replication dynamics, release kinetics, and particle morphology during propagation in MDCK cells. Appl. Microbiol. Biotechnol..

[24] Amato K.A., Haddock L.A., Braun K.M., Meliopoulos V., Livingston B., Honce R., Schaack G.A., Boehm E., Higgins C.A., Barry G.L. (2022). Influenza A virus undergoes compartmentalized replication in vivo dominated by stochastic bottlenecks. Nat. Commun..

[25] Cui P., Shi J., Wang C., Zhang Y., Xing X., Kong H., Yan C., Zeng X., Liu L., Tian G. (2022). Global dissemination of H5N1 influenza viruses bearing the clade 2.3.4.4b HA gene and biologic analysis of the ones detected in China. Emerg. Microbes Infect..

