# Supplementary material for: A Strand-Specific Quantitative RT-PCR Method for Detecting vRNA, cRNA, and mRNA of H7N9 Avian Influenza Virus in a Mouse Model
Source: Viruses. 2025 Jul 17;17(7):1007. doi: 10.3390/v17071007 (PMC12300611; doi:10.3390/v17071007)
Supplement: Supplementary file 1 [file viruses-17-01007-s001.zip › viruses-3716756-supplementary.docx]

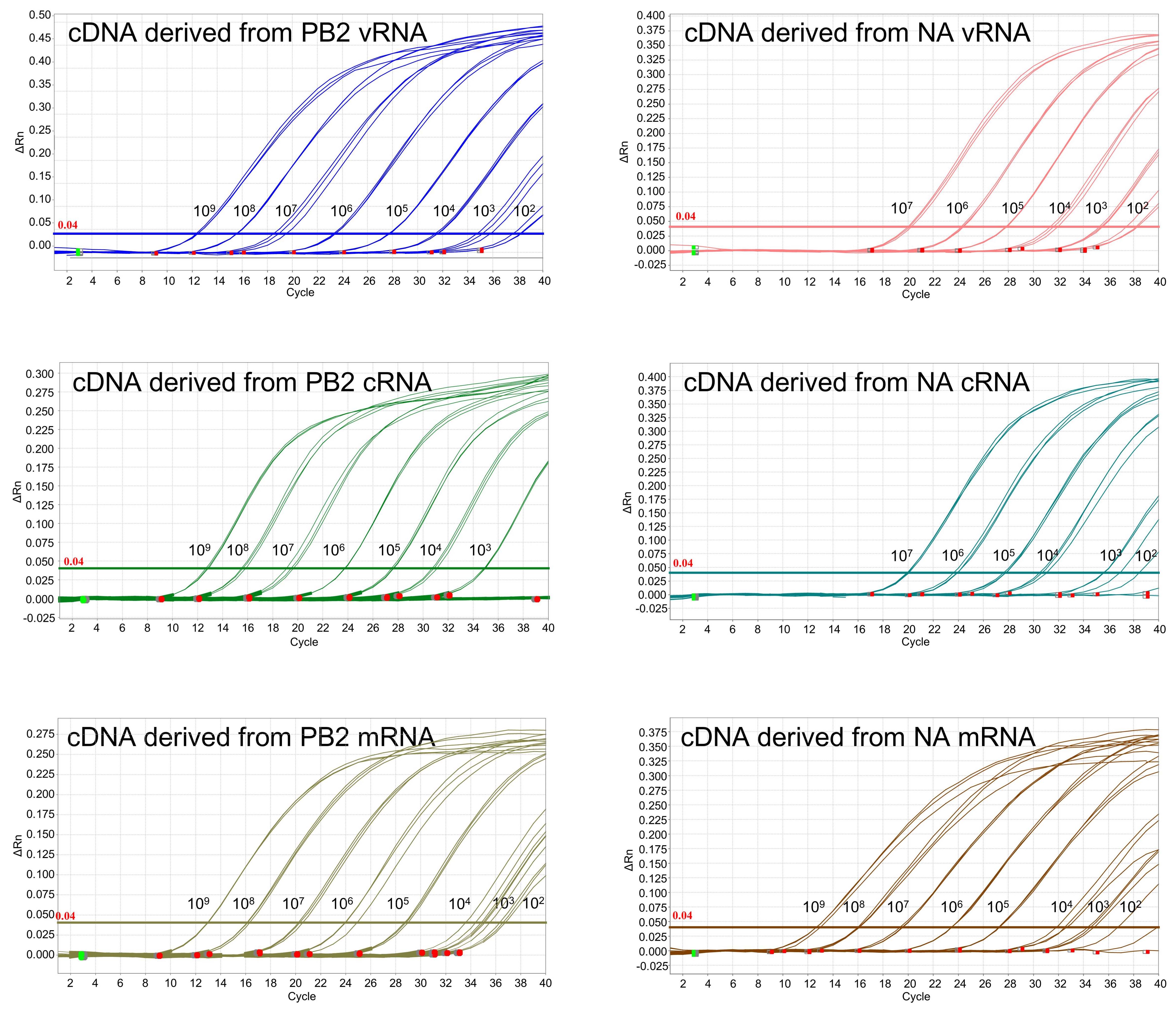


**Figure S1.** Amplification curves of tenfold serial dilutions of cDNA derived from transcribed viral RNA.

**Table S1.** Sequences of TaqMan qRT-PCR primer and probe sets for detection of vRNA, cRNA, and mRNA of the PB2 gene.

| **Target** | **Primers** | **Primer or probe sequences (5’-3’)** | **Purpose** |
| --- | --- | --- | --- |
| PB2 vRNA | PB2vRNAS6RT-TagR | CCCGCTCCCCCAAACAATAACCCTTTCAATCCCTAGTGCC | Reverse transcription of PB2 vRNA |
|  | PB2vRNA-R | TCCGGAACCCCTCACATTCA | TaqMan qPCR of PB2 vRNA |
|  | vRNA-tag | CCCGCTCCCCCAAACAATAA |  |
|  | Probe-PB2vRNA | AGTTAGAGAAGAGAACTGC |  |
| PB2 cRNA | PB2cRNAS1RT-TagR | CTCGGCCAACTCTGATGAAGAGTAGAAACAAGGTCGTTTTTAAAC | Reverse transcription of PB2 cRNA |
|  | PB2cRNA-R | AGGGCAAGGAGACGTTGTGT | TaqMan qPCR of PB2 cRNA |
|  | cRNA-tag | CTCGGCCAACTCTGATGAAG |  |
|  | Probe-PB2cmRNA | ACGGGACTCTAGCATAC |  |
| PB2 mRNA | PB2mRNAS9RT-TagR | CGCGCCTCTTTATTTTTCTCTTTTTTTTTTTTTTTTAAACAATTCGA | Reverse transcription of PB2 mRNA |
|  | PB2mRNA-R | GGGCCAGCATTGAGCATCAA | TaqMan qPCR of PB2 mRNA |
|  | mRNA-tag | CGCGCCTCTTTATTTTTCTC |  |
|  | Probe-PB2cmRNA | ACGGGACTCTAGCATAC |  |

Underline represents different tag sequences.

**Table S2.** Sequences of TaqMan qRT-PCR primer and probe sets for detection of vRNA, cRNA, and mRNA of the NA gene.

Underline represents different tag sequences.

| **Target** | **Primers** | **Primer or probe sequences (5’-3’)** | **Purpose** |
| --- | --- | --- | --- |
| NA vRNA | NAvRNAS6RT-TagR | CCCGCTCCCCCAAACAATAATACTCATTGGAATAGCAAACCT | Reverse transcription of NA vRNA |
|  | NAvRNA-R | AGAGCCCTTTAGTTAAGTTATTGAAATTC | TaqMan qPCR of NA vRNA |
|  | vRNA-tag | CCCGCTCCCCCAAACAATAA |  |
|  | Probe-NAvRNA | TGGATGTTGGTGATGTTT |  |
| NA cRNA | NAcRNAS1RT-TagR | CTCGGCCAACTCTGATGAAGAGTAGAAACAAGGGTCTTTTTCTTC | Reverse transcription of NA cRNA |
|  | NAcRNA-R | GGGACTGCTATCGAGCGTG | TaqMan qPCR of NA cRNA |
|  | cRNA-tag | CTCGGCCAACTCTGATGAAG |  |
|  | Probe-NAcmRNA | CAGGAATTCTGTACTGGAACA |  |
| NA mRNA | NAmRNAS9RT-TagR | CGCGCCTCTTTATTTTTCTCTTTTTTTTTTTTTTTTCTTCATCTTAG | Reverse transcription of NA mRNA |
|  | NAmRNA-R | GAAGGGGACTGCTATCGAG | TaqMan qPCR of NA mRNA |
|  | mRNA-tag | CGCGCCTCTTTATTTTTCTC |  |
|  | Probe-NAcmRNA | CAGGAATTCTGTACTGGAACA |  |

**Table S3.** Strand-specific reverse transcription system of PB2 or NA vRNA.

| 10 × RT Mix | 2 µl |
| --- | --- |
| RT-primer | 0.5 µl |
| total RNA (2 µg) | 5 µl |
| HiScript II Enzyme Mix | 2 µl |
| RNase-free ddH_2_O | 10.5 µl |
| Total | 20 µl |

**Table S4.** Strand-specific reverse transcription system of PB2 or NA cRNA and mRNA.

| 10 × RT Mix | 2 µl |
| --- | --- |
| RT-primer | 0.5 µl |
| RNA (2 µg) | 5 µl |
| HiScript II Enzyme Mix | 2 µl |
| Saturated trehalose solution | 10.5 µl |
| Total | 20 µl |
